# Effects of Caloric Restriction Diet on Arterial Hypertension and Endothelial Dysfunction

**DOI:** 10.3390/nu13010274

**Published:** 2021-01-19

**Authors:** Nicola Di Daniele, Giulia Marrone, Manuela Di Lauro, Francesca Di Daniele, Daniela Palazzetti, Cristina Guerriero, Annalisa Noce

**Affiliations:** 1 UOC of Internal Medicine-Center of Hypertension and Nephrology Unit, Department of Systems Medicine, University of Rome Tor Vergata, Via Montpellier 1, 00133 Rome, Italy; giul.marr@gmail.com (G.M.); dilauromanuela@gmail.com (M.D.L.); francesca.didaniele@gmail.com (F.D.D.); daniela.palazzetti96@gmail.com (D.P.); cristinaguerriero@hotmail.it (C.G.); annalisa.noce@uniroma2.it (A.N.); 2 School of Applied Medical, Surgical Sciences, University of Rome Tor Vergata, Via Montpellier 1, 00133 Rome, Italy

**Keywords:** arterial hypertension, endothelial dysfunction, organ damage, caloric restriction diet, intermittent fasting

## Abstract

The most common manifestation of cardiovascular (CV) diseases is the presence of arterial hypertension (AH), which impacts on endothelial dysfunction. CV risk is associated with high values of systolic and diastolic blood pressure and depends on the presence of risk factors, both modifiable and not modifiable, such as overweight, obesity, physical exercise, smoking, age, family history, and gender. The main target organs affected by AH are the heart, brain, vessels, kidneys, and eye retina. AH onset can be counteracted or delayed by adopting a proper diet, characterized by a low saturated fat and sodium intake, a high fruit and vegetable intake, a moderate alcohol consumption, and achieving and maintaining over time the ideal body weight. In this review, we analyzed how a new nutritional approach, named caloric restriction diet (CRD), can provide a significant reduction in blood pressure values and an improvement of the endothelial dysfunction. In fact, CRD is able to counteract aging and delay the onset of CV and neurodegenerative diseases through the reduction of body fat mass, systolic and diastolic values, free radicals production, and oxidative stress. Currently, there are few studies on CRD effects in the long term, and it would be advisable to perform observational studies with longer follow-up.

## 1. Introduction

As is well known, high blood pressure (BP) is one of the most important public health problems worldwide. High BP values may be caused by elevated cardiac output, enhancement of peripheral vascular resistance, or by a combination of both, and they negatively impact on the average life expectancy [1,2]. In fact, it is estimated that the prevalence of arterial hypertension (AH) among adults will increase from 26.4% to 29.2% in the year 2025. Currently, the World Health Organization (WHO) states that it affects one in four men and one in five women, meaning more than 1 billion people [3]. Therefore, cardiovascular (CV) diseases are one of the main causes of death in the word, together with newly arising infectious causes such as SARS-CoV-2 [4]. The most frequent CV diseases are myocardial infarction and stroke [5]. The affected target organs by AH are heart, brain, kidneys, vessels, and eye retina [3]. According to the degree of AH, clinicians should provide the most appropriate pharmacological and non-pharmacological therapy, as well [6]. The latter include undertake a healthy lifestyle, practice habitual physical exercise, avoid smoke, follow a diet with low-sodium intake (<100 mEq/day), and lose weight (in case of overweight or obese subjects). If lifestyle changes are not able to normalize BP, it is mandatory to begin a pharmacological treatment in order to restore BP values in the range of normality [7].

Currently, it is speculated that one of the possible nutritional strategies useful for the management of AH is a caloric restriction diet (CRD) [8,9].

Numerous studies have shown that eating habits are able to modify CV risk factors [10,11,12]. These impact on endothelial function, favoring the inflammatory processes underlying atherosclerosis [13]. In physiological conditions, the vascular endothelium maintains its tone through the release of signaling molecules with vasodilator (such as nitric oxide- (NO)) and vasoconstrictor (such as angiotensin II) action [14]. The endothelial dysfunction occurs when there is an abnormal production of reactive oxygen species (ROS) of pro-inflammatory cytokines, such as interleukin (IL)-1 and tumor necrosis factor (TNF)-α, and a decrease release of NO [15]. This condition triggers the atherosclerosis process [14]. For this reason, it is important, if not essential, undertake a nutritional treatment in patients with high BP. In this review, we focused on the possible beneficial effect of CRD on the BP control, highlighting the main antihypertensive mechanisms exerted by this nutritional approach.

## 2. Definition, Classification, and Management of Arterial Hypertension

AH is defined as a “condition characterized by increased blood pressure in the blood vessels” and when it becomes too high, and persists over time, it can damage the arteries and organs, leading the heart to a greater cardiac output [16]. Therefore, a condition in which systolic BP (SBP) is higher than 140 mm Hg and/or diastolic BP (DBP) is more than 90 mm Hg is defined as AH, as reported by the latest 2018 European Society of Cardiology (ESC) and European Society of Hypertension guidelines (ESH) [17]. 

Specifically, BP is optimal when SPB values are <120 mm Hg and DBP values are <80 mm Hg, and it is normal when SPB values are between 120 and 129 mm Hg and DBP values are between 80 and 84 mm Hg. BP is considered high-normal for SBP values between 130 and 139 mm Hg and for DPB values between 85 and 89 mm Hg. It defines grade 1 AH when SPB values are between 140 and 159 mm Hg and/or DPB values are between 90 and 99 mm Hg; grade 2 AH when SPB values are between 160 and 179 mm Hg and/or DPB values are between 100 and 109 mm Hg; grade 3 AH when SPB values are ≥180 mm Hg and/or DPB values are ≥110 mm Hg [17].

**Figure 1:**
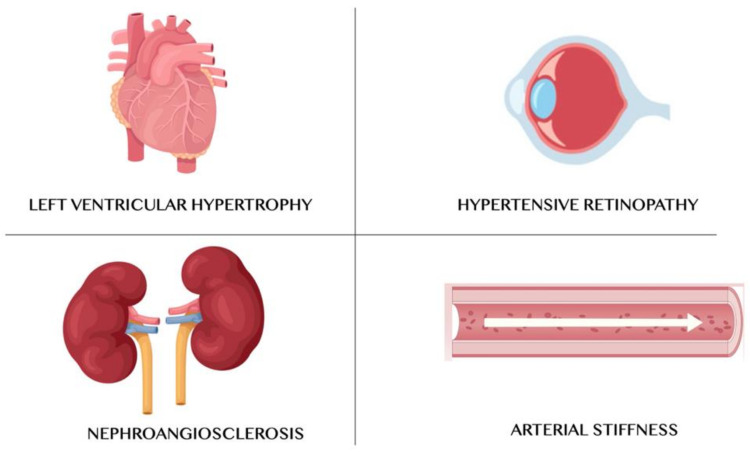
Main target organs of arterial hypertension.

High BP can cause left ventricular hypertrophy and then heart failure, renal failure (nephroangiosclerosis), accelerated atherosclerosis, and hypertensive retinopathy (Figure 1). AH arises from the combination of genetic and environmental factors. Therefore, it should be useful to identify predisposed patients and instruct them to lifestyle changes. Such lifestyle changes concern to the adoption of healthy diet, in order to avoid high alcohol consumption, to promote regular physical activity, to maintain the normal BW, to stop smoking and to avoid passive smoking [18]. In Italy, it is estimated that there are about 15 million hypertensive subjects; in fact, 28.3% of the population is affected by AH [19,20]. Several clinical trials have shown that a lifestyle change can delay or prevent AH onset in people who are not hypertensive, delay or prevent drug therapy in subjects with grade I AH, contribute to the BP reduction in hypertensive individuals already in pharmacological therapy, and reduce the number and dosage of antihypertensive drugs [21]. 

The wide availability of drugs offers the possibility to obtain a fast-hypotensive effect and to act positively on the mechanisms that predispose to CV events. 

## 3. Arterial Hypertension and Endothelial Alterations

AH is the most important risk factor for CV diseases [22]. In fact, vascular alterations (such as endothelial dysfunction) are a pathophysiological response mechanism to the development of organ damage, and they should be taken into account for the global CV risk assessment. The endothelium plays a pivotal role, and it consists of 1.2 billion cells, with a weight exceeding 1.5 kg and an area of 400 m^2^ [23]. It forms a thin cellular lamina in direct contact with the bloodstream, representing the innermost layer of the vessel wall. The most important functions performed by endothelium are the modulation of the inflammation, the regulation of the vasomotor tone, the promotion and inhibition of cell proliferation and the modulation of the coagulative cascade [24,25,26]. The main physiological mediator of endothelium is NO, but it displays also other important functions at the level of the central nervous and immune systems. Endothelial cells produce NO through the enzyme NO-synthase (NOS), which transforms L-arginine amino acid into citrulline [27]. The activity of NOS is stimulated by numerous mediators, such as bradykinin and acetylcholine, or by mechanical forces, mainly “shear stress” [27]. NO is a volatile gas that has few seconds half-life and that, spreading towards the vessel wall smooth muscle cells, causes the release of cyclic guanosine-monophosphate (cGMP) with the consequent reduction of intracellular calcium [28]. 

The term “endothelial dysfunction” identifies a pathophysiological condition characterized by anatomically intact endothelial cells, but when stimulated, instead of solely determining the production of NO, activates in parallel the production of ROS, which causes the degradation of NO [29]. Although the endothelial dysfunction is mainly caused by increased destruction of NO, it may also depend on its reduced production due to L-arginine substrate deficiency, or by vasoconstriction induced by factors derived from cyclooxygenase [23]. 

The endothelial damage is instead represented by the destruction of endothelial cells, where the regeneration of these cells is difficult to achieve [30]. The BP increase causes an enhancement in the production of superoxide and a decrease in the bioavailability of NO [31]. There is also another mechanism involving the renin–angiotensin–aldosterone system (RAAS). The angiotensin conversion enzyme (ACE) acts on the endothelium by converting angiotensin I to angiotensin II, an endocrine vasoactive peptide. The latter is an active protein able to induce vasoconstriction through the calcium-dependent myosin phosphorylation with the contraction of arterial smooth cells, inducing an enhancement of BP levels. Moreover, angiotensin II stimulates in the kidneys the sodium reabsorption which, in turn, induces water retention. Angiotensin II promotes also the production of endothelin, a class of proteins with paracrine/vasoconstrictive action, causing an increase in blood pressure [32,33]. Endothelin is synthetized in the endothelial cells through two different pathways. The first is named “constitutive”, and it is characterized by a continuous release of endothelin from macrovesicles which, in turn, interact with their own receptors, maintaining the vascular tone. The second is defined as a “regulated” mechanism and is activated by external physiological or pathophysiological stimuli. In fact, the endothelin that is released by the endothelial cells through this system, interacts with two types of receptors, namely, ET_A_, placed on the smooth muscle layer of the vessel, and, to a lesser extent, with ET_B_, which mediate the vasoconstrictive action [34].

Normally, there is a balance between vasoconstrictive and vasodilating substances in the bloodstream, but in the case of AH, the bioavailability of endothelin can be increased in parallel with a reduction in the bioavailability of NO. Angiotensin conversion enzyme inhibitors (ACE-I) and angiotensin receptor blockers (ARB) represent the first-line therapies for AH [7,17]. Dysfunctional endothelium shows a poor vasomotor function, which leads to an elevated BP at rest [35]. To date, it is speculated that there is a correlation between AH and endothelial dysfunction, but the issue is still unresolved due to the few studies in this regard [36,37,38,39].

## 4. Methods

PubMed, Web of Science, and Scopus online libraries were searched up until November 2020 in order to assess the most interesting and recent evidences about CRD and AH. The search was performed manually, using a combination of MeSH terms and keywords such as “caloric restriction diet”, “arterial hypertension”, “endothelial dysfunction”, and “dietary protocol”. All the studies were in the English language.

## 5. Caloric Restriction Diet

The CRD innovative approach consists of a chronic reduction in daily caloric intake of about 25–30% compared to the normal caloric intake, without any exclusion of food groups [40]. Although this regimen is not standardized, numerous studies show its effectiveness. Currently, according to the Calorie Restriction Society, subjects who follow a self-imposed CRD regimen are characterized by an increase in life expectancy. This regime consists of a caloric restriction with a daily consumption lower than 1800 kcal for an average period of 15 years and with an energy intake 30% lower than a group of individuals (homogeneous for age, gender, and socio-economic status) who consumed a Western diet model [40,41].

The first animal study to assess the beneficial effects of the CRD was carried out in rats in the 1900s [42]. Following studies showed that restricting food intake in mice delayed their growth but also extended their lifespan by to two times. Compared to a group that did not follow any restrictions, the CRD mice group showed anti-aging effects [43]. The most reliable hypothesis of the anti-aging effect of CRD is associated with reduced oxidative stress (OS). During the cellular respiration, oxygen is converted into ROS. ROS are able to react with macromolecules within cells causing the process of cellular aging. Therefore, CRD was initially designed for its antioxidant effect and consequently for its anti-aging action [44]. At this regard, numerous studies have been carried out, although contrasting results have been obtained. In fact, in some mice, without the superoxide dismutase enzyme, there was not increase in the aging, despite the enhanced of oxidative damage [45,46]. Animal studies on CRD showed that initial food restriction followed by alternating fasting acted positively on glycemic control, body weight (BW) reduction, insulin sensitivity, and BP control [47]. Anisimov and Bartke confirmed that serum values of glycemia and insulin were lower under CRD treatment [48]. In this context, the key role exerted by CRD is due to the decrease in insulin and insulin-like growth factor-1 (IGF-1) release and to the insulin sensitivity enhancement, observed both in rodents and in monkeys [49,50]. The results obtained in these studies confirm that the aging-related pathologies’ arose later in the CRD group compared to *ad libitum* diet group [51].

In recent years, many theories have been developed to explain the CRD beneficial effects. Most of them focused their attention on the sirtuin family. Sirtuins are proteins nicotinamide dinucleotide (NAD+)-dependent deacylases able to prevent some diseases and to modulate several aspects of cellular aging, promoting DNA integrity through the maintenance of the normal degree of chromatin condensation and repairing promptly DNA damage [52,53,54,55]. In mammals, sirtuin1 (SIRT1) cytoplasmatic protein acts on the control of cell cycle, particularly on the apoptosis and on the mitochondrial metabolism [56]. Several studies showed that sirtuins levels after CRD increased in some specific tissues such as the gut, brain, kidney, adipose tissue, and skeletal muscle [57,58,59]. In a study conducted by Meyer et al., the authors demonstrated a significant effect of CRD on cardiovascular aging [60]. Twenty-five healthy subjects (mean age 53 ± 12 years) that followed CRD were matched with 25 subjects homogeneous for age and gender (control group) that followed a typical Western diet. All enrolled subjects underwent to diastolic function evaluation by transmitral flow, Doppler tissue imaging, and model-based image processing (MBIP) of E waves and inflammatory status assessment thanks to C-reactive protein (CRP), TNF-α, and transforming growth factor-beta1 (TGF-β1) blood sampling. Results showed that parameters such as Doppler flow diastolic function mean indexes, BP, CRP, TNF-α, and TGF-β1 were significantly lower in the CRD subject, compared to the control group. The results of this study highlighted that CRD is able to ameliorate cardiac function, slowing the cardiac-aging process and decreasing systemic inflammation and BP values. Furthermore, it is confirmed that CRD increases life expectancy.

Following a CRD, it is possible counteract aging in all living forms, lengthening both the median and the maximum duration of life, and delaying over time the appearance of CV and neurodegenerative diseases [61,62].

Human’s life span has increased considerably due to the improvement of hygienic conditions and greater availability of pharmacological therapies. Taking into account the idea that the caloric restriction prolongs the life span, the mechanism of action by which this is possible, is not fully understood yet. Recent studies have shown that CRD can determine the damaged DNA repair and decrease fat mass, SBP and DBP values, the production of free radicals. The results obtained from the CRD can occur quickly, but they can mitigate in case of its suspension [63].

## 6. Caloric Restriction Diet and Arterial Hypertension

**Figure 2:**
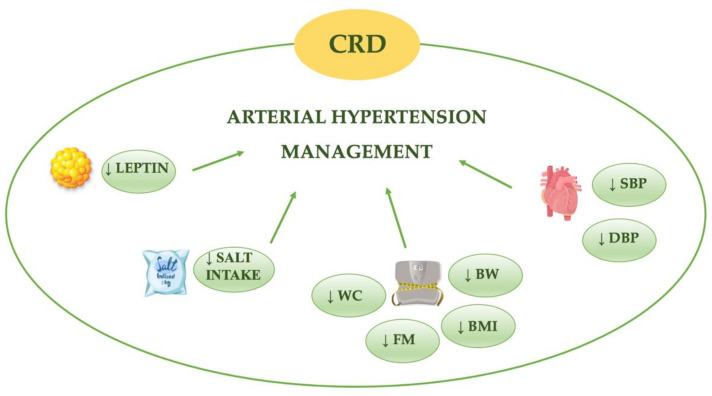
Beneficial effects of caloric restriction diet (CRD) on arterial hypertension. BMI, body mass index; BW, body weight; DBP, diastolic blood pressure; FM, fat mass; SBP, systolic blood pressure; WC, waist circumference, ↓ decrease.

The CRD would seem to exert a beneficial effect against AH (Table 1) and for this reason represents a useful tool for its clinical management (Figure 2).

An important study conducted in this regard was the CALERIE (Comprehensive Assessment of Long-term Effects of Reducing Intake of Energy) [85]. The CALERIE study was a randomized controlled trial with a two-year follow up. This study was divided in two phases: CALERIE-1 and CALERIE-2 [73,75]. CALERIE-1 study was performed to assess the possible effects induced by a reduction of 10-30% of caloric intake on body composition parameters and lipid profile after 6 and 12 months in a population of middle-aged non-obese subjects. CALERIE-1 results showed an improvement in lipid and glycemic profile and a reduction in BW and fat mass. CALERIE-2 was the largest multi-center study on CRD, involving three centers, namely the Pennington Biomedical Research Center (Baton Rouge, LA, USA), Tufts University (Boston, MA, USA), and Washington University School of Medicine (St. Louis, MO, USA), and was coordinated by Duke Clinical Research Institute (Durham, NC, USA). A total of 2020 subjects were enrolled randomly with a 2:1 allocation into two subgroups: 145 in the CRD group and 75 in the *ad libitum* group. The CRD group followed 25% caloric restriction for two-years [78]. After two years of diet treatment, cardiometabolic risk factors such as low-density lipoprotein cholesterol (LDL-c), total cholesterol/high-density lipoprotein cholesterol (HDL-c) ratio, SBP and DBP decreased. Moreover, serum biomarkers such as CRP, insulin sensitivity index and metabolic syndrome score were reduced. Moreover, BW was significantly lower in the CRD group when compared to the *ad libitum* group (average weight loss in CRD group was 7,5 kg *vs* average BW increase of 0,1 kg in *ad libitum* group). These data showed that a period of two–years of CRD was able to decrease cardiometabolic risk factors in middle-aged non-obese subjects. For this reason, it is possible to consider CRD as nutritional therapeutic approach to enhance life expectation and reduce the onset of chronic non-communicable diseases such as diabetes mellitus, cancer, chronic kidney disease, and AH, among others [86,87].

Other studies have been conducted to investigate the role of CRD in the control of AH. In particular, a study performed by Most et al. on caloric restriction (25%), with two years follow–up, evaluated the possible reduction of CV risk factors and insulin resistance in non-obese subjects and whether the results obtained were maintained over time or were limited to the period study. The authors showed a significant weight loss associated to a decrease in SBP and DBP and an improvement in other parameters, such as lipid profile and insulin resistance. These improvements, with the exception of insulin sensitivity, appeared to be maintained over time [77].

Another study examined the impact of CRD (25%) for six–month follow-up period in patients affected by type 2 diabetes mellitus, AH, and glomerular hyperfiltration. The results were positive, since the authors observed that the CRD reduced glomerular hyperfiltration and, improved insulin sensitivity and SBP and DBP values, compared to the control the group that followed a standard diet. In general, it is speculated that CRD ameliorates CV risk factors [76]. To explain the mechanism underlying the reduction in BP induced by CRD, it has been hypothesized that it may act through the activation of the autonomic nervous system. This hypothesis was investigated by Nakano et al, who observed a reduction in SBP and DBP in obese hypertensive patients in CRD treatment (800 kcal/day) with normal sodium content for two weeks [71]. In obese individuals, CRD would appear to improve the balance of night activation between the vagal/sympathetic system. In fact, in obese subjects, there is an alteration of the autonomic control of the heart due to a prevalence of the sympathetic component over the parasympathetic component one in the autonomic equilibrium. A subsequent study confirmed the effects of short-term CRD (three weeks) in combination with a high–intensity exercise program on heart rate variability (HRV) in normotensive obese subjects, demonstrating that a short-term program of CRD associated with high intensity exercise can positively change the autonomic profile, leading to a reduction in HRV and an increase of parasympathetic activity [72]. The long-term effects of CRD on autonomic nervous system activity are relevant. In an animal study, long-term CRD intake would appear to be able to slow down age-related functional changes in the autonomic system. In fact, in male rats, CRD causes an increase in HRV of the highest frequency components (biomarkers of parasympathetic nervous system activity) and reduces the low-frequency component of diastolic pressure variability (biomarkers of sympathetic tone) [84].

## 7. Caloric Restriction Diet and Hypertensive Organ Damages

**Figure 3:**
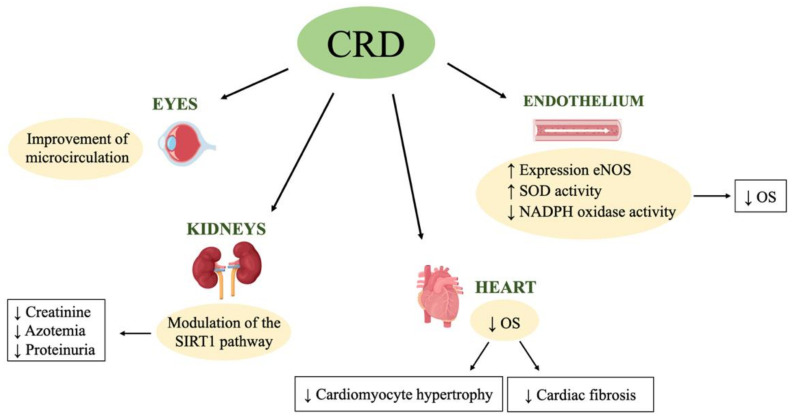
Improvement of caloric restriction diet on hypertensive organ damage. CRD, caloric restriction diet; eNOS, endothelial nitric oxide synthase; NADPH, nicotinamide adenine dinucleotide phosphate hydrogen; OS, oxidative stress; SIRT1, sirtuin 1; SOD, superoxide dismutase, ↑ increase, ↓ decrease.

The damage caused by AH is directed to important target organs and develops essentially as a direct and/or indirect consequence of vascular pathology (Figure 3) [88]. In the majority of patients with high BP, the increased values may not be too severe, and the direct damage may develop after years of the AH onset. As previously mentioned, the organs most affected by AH are the heart, kidneys, eye retina, vessels and brain. The pathogenesis of organ damages is complex and varied [89].

Increased BP, in addition to the direct damage to the vessel walls, can cause additional pathogenic pathways that include endothelial dysfunction, inflammation, and OS, as well as, all the changes that induce vascular structure alteration, namely arterial rigidity, already underlining to the aging. Therefore, AH is considered to be an accelerated form of cardiovascular aging. Several studies have highlighted a protective action exerted by CRD against hypertensive organ damage.

### 7.1. Left Ventricular Hypertrophy

Over time, in hypertensive patients, the left ventricular becomes increasingly rigid and diastolic filling is compromised. Left ventricular hypertrophy (LVH) can be described as the thickening of the ventricular wall caused to cope with the overload imposed to the heart for offset peripheral resistances, hence the increase in BP. LVH may occur in both sexes and leads to an increase in either oxygen consumption or energy expenditure of cardiac output [90,91].

It has recently been observed that in hypertensive subjects, LVH is related to hemodynamic and non-hemodynamic mechanisms, which are observed in particular in obese and overweight subjects. In fact, LVH is observed mainly in these patients. The non-hemodynamic mechanisms involve an impairment of lipid metabolism but also the production by adipose tissue of adipokines, such as adiponectin, leptin, and TNF-α [92]. In confirmation of this, recent animal studies have demonstrated that leptin-deficient and leptin-resistant mice exhibit obesity, insulin resistance, and LVH, although the mechanisms that related LVH and alteration of leptin metabolism are not fully understood [93]. Most obese and hypertensive subjects show leptin resistance, and their leptin plasma level is directly related to cardiac hypertrophy [94]. Leptin is involved in heart complications typical of obesity, including AH. It has been shown that an acute increase in leptin does not appear to have any effect on the BP values. On the contrary, a chronic enhancement in leptin increases BP values, stimulating the sympathetic nervous system and simultaneously altering the mechanisms designed to counteract it, such as the natriuresis and the synthesis of NO [95]. Finally, chronic hyperleptinemia appears to be directly related with BP levels [96].

However, leptin may play also a cardioprotective role, related to BW reduction and improvement of myocardial metabolism. Leptin avoids an excessive accumulation of lipids in the heart in obese subjects and inhibits the formation of toxic lipid derivatives, which induce a condition called “cardiac lipotoxicity” [97]. This cardioprotective role exerted by leptin in obese patients, was confirmed only in a study conducted on 1172 black obese women, which revealing an inverse correlation between leptin and LVH severity [98]. 

CRD is capable of reducing leptin blood levels [74]. This reduction would initially seem to be related to the degree of caloric restriction, but in the long time it appears to be related to the reduction of BW and visceral fat [70]. The CRD seems to exert a cardioprotective action derived from the decrease of OS and inflammation, from the cellular regulation of iron homeostasis and from cardiac remodeling. For these reasons, CRD is definitely a new adjuvant treatment, in combination with pharmacological therapy, for cardiomyopathy in hypertensive patients [99].

Moreover, CRD helps to improve the picture of cardiac hypertrophy through SIRT1 and peroxisome proliferator-activated receptor gamma coactivator 1(PGC1)-α pathways [68]. In fact, as previously reported, CRD would seem to increase the activity of SIRT1, which in turn stimulates PGC1-α, involved in inflammatory signaling pathways. The protective action induced by SIRT1 is related to glucose metabolism as it counteracts the accumulation of cardiac fatty acids, exerting a protective action against cardiotoxicity [68]. Another CRD cardioprotective effect is related to suppression of OS present in AH condition. In fact, recent studies show how ROS production is involved in cardiomyocyte hypertrophy and cardiac fibrosis [100]. Kobara et al., demonstrated that CRD reduces ROS production at the cardiac level, improving the condition of OS and ameliorating cardiac hypertrophy and fibrosis in hypertensive mice, subjected to a 40% reduction in caloric intake compared to the control group, after four weeks [67]. Finally, the last cardioprotective effect induced by CRD is related to the regulation of cellular iron homeostasis. The authors observed a reduction in inflammation, OS, and LVH, associated with a normalization of iron overload in leptin-resistant obese mice subjected to 12 weeks of CRD. This effect would appear to be due to the modulation of gene expression induced by CRD of the genes involved in iron homeostasis at the level of heart tissue [69].

Further studies will be needed for demonstrate if factors such as age and period of following CRD impact on obtained results.

### 7.2. Kidney Damage—Nephroangiosclerosis

Classically, the term nephroangiosclerosis (NAS) refers to the presence of non-immune-mediated vascular lesions and it is a very common condition in presence of high BP values [101]. The organ damage caused by AH on the renal parenchyma shows up by the increase in plasma creatinine and in early stage by the presence of albuminuria [102]. The classic definition has been revised as the immune cells contribute to determining kidney organ damage. In fact, it has been highlighted how different stimuli, including an increase in angiotensin II production or a diet with a high salt content, can induce a response by the immune system that causes an inflammatory reaction mainly at the level of the organs involved in the control of BP values (such as kidneys, heart and nervous system) [103,104,105]. This observation is confirmed by the fact that hypertensive patients usually present a low-grade chronic inflammatory state. It has also been shown that a high salt diet can stimulate cells of the immune system, including T lymphocytes, which in turn produce pro-inflammatory cytokines, while the activation of monocytes and macrophages can induce both vasoconstriction and renal sodium retention [106]. The three mechanisms described above, taken together, contribute to producing organ damage at the level of the kidney, such as NAS [107]. In terms of NAS, it is important to reduce BP values and proteinuria since they are related to the progression of the nephropathy and to cardiovascular events [108].

According to a recent meta-analysis that examined 27 studies, CRD appears to exert a protective effect against renal damage induced by AH [109]. This meta-analysis demonstrated that in chronic kidney disease (CKD) rat models undergoing CRD, there was a higher reduction in BP, creatinine, azotemia, and proteinuria, compared to a control group fed *ad libitum* [109]. The authors also demonstrated a survival rate increase at 700-800 days. This nephroprotective effect of CRD would seem to be exerted through the activation of AMP-activated protein kinase (AMPK) and the modulation of the SIRT1 pathways, the latter having been previously described [110]. In particular, the CRD seems to impact, in CKD rat models, on the activity of the AMPK and of the mammalian target of rapamycin (m-TOR) pathways, both implicated in cellular energy metabolism. Increased expression of m-TOR has been shown to accelerate kidney aging, while phosphorylation of AMPK inhibits this process. A short-term, CRD has been seen to induce an up-regulation of AMPK and a down-regulation of m-TOR, resulting in slowing the aging process of renal tubular cells [111]. Therefore, CRD in combination with a reduced sodium intake could be a valid nutritional alternative in the treatment of NAS patients.

In fact, a meta-analysis conducted by D’Elia et al. [112] confirmed the nephroprotective role of sodium restriction, pointing out the relation among dietary sodium restriction and urinary albumin excretion (UAE), a biomarker of CKD progression. The authors considered a total of 11 studies who agreed on the close association between the reduction of UAE induced by a decrease of dietary sodium intake. Moreover, as previously demonstrated in the literature, the authors confirmed that this nutritional approach could ameliorate the therapy based on RAAS-blocking drugs in hypertensive patients. In this perspective, the reduction of UAE induced by low dietary sodium intake could impact on slowing down the progression of CKD, rather than reducing cardiovascular morbidity and mortality.

### 7.3. Arterial Stiffness

Arterial stiffness (AS) is an important CV risk factor [108], and it induces the rigidity of the arterial wall [113,114]. BP is the main determinant of AS, and the aortic rigidity is accentuated by other concomitant diseases such as diabetes mellitus, metabolic syndrome, CKD, and obesity [115]. 

The aortic pulsating wave velocity (PWV) correlates with the presence of organ damage and in the heart, an increased AS induces ventricular afterload, ventricular hypertrophy, and reduced coronary perfusion [116]. Sustained increases in BP promote collagen matrix synthesis, causing subsequent vascular thickening and vasal stiffening [117]. 

The protective role of CRD on AS has been extensively studied. Specifically, CRD seems to exert a protective effect on the endothelium through an antioxidant action and thanks to an increased NO bioavailability [64]. Rippe et al. observed an improvement in carotid artery endothelium-dependent dilation mediated by the enhancer of the expression of endothelial nitric oxide synthase (eNOS) in mice undergoing CRD (approximately 30% of caloric restriction) compared to the control group (fed *ad libitum*) after 8 weeks [65]. Similar results were observed in a subsequent study, where the authors demonstrated a reduction in nicotinamide adenine dinucleotide phosphate hydrogen (NADPH) oxidase activity and an increase in superoxide dismutase (SOD) and in catalase activities, confirming that the CRD has an important antioxidant action, in animal models [66]. The effect on the increase of NO availability would seem to be exerted by the action of CRD on SIRT1, which deacetylases and activates eNOS and increases the expression of antioxidant genes [118].

A recent meta-analysis [119] examined 11 clinical trials on the correlation between dietary salt restriction and AS. In the analyzed studies, an average decrease in dietary salt restriction highlighted a 2.38% reduction of PWV. This data would seem of great interest, but there are some concerns, common to all the studies examined, such as the duration of the intervention (ranging from a minimum of 1 to a maximum of 6 weeks) and the small samples of enrolled subjects in each study.

### 7.4. Hypertensive Retinopathy

Hypertensive retinopathy is an ocular disease involving the arteries and veins of the retina, the optic nerve, and the choroid. The cause of this condition is AH. In subjects that have high BP values, over the time, a modification of the retinal arteries takes place, which tends to shrink, and the retinal veins instead tend to assume a different course, no longer linear. These changes can impair normal vision and lead to the formation of ischemic areas of the retina, with the formation of exudates [120]. In more advanced forms, the vision can be compromised and become blurred or distorted. The diagnosis is made by examining the ocular fund which allows the physician to assess the vision, or to see if there are small bleeding or edema. Depending on what can be observable, it can be possible to evaluate the stage of the disease. When AH is associated with diabetes mellitus, both increase the risk of CV complications and the retinopathy becomes more severe. Since the retina is one of the organs most sensitive to changes in microcirculation, signs of damage to this organ have a high prognostic value for myocardial ischemia, carotid sclerosis and coronary artery damage [121].

The role of CRD on the eye has been extensively investigated, especially with regard to age-related eye diseases [122], but no studies have been conducted to investigate the *in vivo* effect of CRD on hypertensive retinopathy. However, as reported above, it is well known that the control of BP within the normal range, also through CRD, exerts a preventive role against the onset of eye microvascular abnormalities leading to hypertensive retinopathy [123].

## 8. Secondary AH: Traditional Dietary Nutritional Protocols

To date, secondary AH affects about 5-10% of the hypertensive subjects. In the greater percentage of these cases, it is possible to observe reversible causes [124]. In the population over 65 years of age, the etiology of secondary AH is due to conditions such as renal artery stenosis, CKD, hypothyroidism, hyperaldosteronism, obstructive sleep apnea, and Cushing syndrome [125]. Resistant AH is a condition characterized by high levels of BP (i.e. >140/90 mmHg in the general population and 130/80 mm Hg in diabetic or nephropathic patients) that could not be controlled by medications (for example the combination of three or more antihypertensive drugs belonging to different classes, including diuretics at their maximum tolerated dosage), according to current guidelines [126].

A large number of scientific evidences supports the notion that multiple dietary factors influence BP levels in the general population both in cases of primary and secondary AH [127]. Secondary forms of AH are relatively rare, but their recognition is very important if we consider that some of them can heal permanently after the removal of the cause.

**Figure 4:**
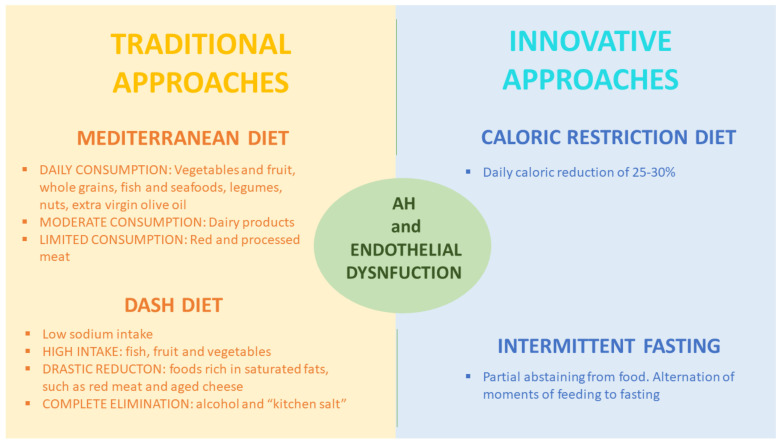
Traditional vs. innovative nutritional approaches for arterial hypertensions and endothelial dysfunction treatment. AH, arterial hypertension.

The suspicion of a AH secondary form should raise if (i) AH is resistant to a triple drug therapy that includes a diuretic drug, (ii) AH, characterized by particularly high BP values, diagnosed in young people (about 30 years of age), especially in women, (iii) in case of accelerated or malignant hypertension (renovascular hypertension), (iv) AH becomes suddenly uncontrollable with poly-pharmacological therapy or (v) there is an excessive BP values reduction after administration of RAAS blocking drugs [128]. For the prevention and treatment of secondary AH, as well as for the primary, is used a dietary model similar to the Mediterranean diet called DASH diet is used, which stands for “Dietary approaches to Stop Hypertension”. The DASH diet was developed by studies supported by the U.S National Institute of Health (NIH), as a nutritional approach for the treatment of AH that could be of valuable assistance in terms of reaching optimal BP values, with minimal use of antihypertensive drugs (Figure 4) [129]. The purpose of the DASH diet is to restore homeostasis in all AH patients with an incorrect and unhealthy lifestyle. The DASH diet is considered to be the dietary “gold standard” approach by the American Society of Hypertension in reducing multiple CV risk factors, related to primary and secondary AH [130]. The DASH diet is characterized by a high intake of fish, fruit, and vegetables; a drastic reduction of the consumption of foods rich in saturated fats, such as red meat and aged cheeses; and a complete elimination of alcohol and “kitchen salt” assumption. While CRD, previously discussed, focuses on the number of calories consumed, reducing them by 25–30%, the DASH diet mainly focuses on the quality of micro- and macro-nutrients assumed. Numerous studies have shown that the DASH diet induces a greater reduction in BP values than other dietary interventions or physical exercise programs [131]. 

Most of the studies on salt reduction and weight loss were carried out on middle-aged subjects. In particular, the Trial of Nonpharmacological Interventions in The Elderly (TONE) study showed that, in AH subjects, a moderate salt restriction and weight loss allow the reduction of the number and dosage of antihypertensive drugs [131].

The study carried out by Harsha et al. on 459 adults with SBP values lower than 160 mm Hg and DBP values between 80 and 95 mm Hg demonstrated that the DASH diet reduced BP and represent a nutritional approach to prevent and treat AH [79].

For this reason, the Joint National Committee for Prevention, Treatment and Evaluation of High Blood Pressure strongly recommends following this dietary-nutritional approach to counteract high BP values [132]. 

Moreover, a recent meta-analysis conducted by D’Elia et al. [133] examined the impact of dietary sodium restriction on central blood pressure (cBP). In fact, if the effects of sodium restriction on peripheral BP are well known, its effects on cBP are scarce. The authors found a statistically significant association between the reduction of BP and central pulse pressure, speculating that sodium restriction also impacts on central BP values. For this reason, a diet with a low sodium intake is a useful tool to counteract the onset and/or the progression of CV disease, especially in normotensive subjects and in prehypertensive patients. This effect supports previous studies that exhort the benefits of a low sodium intake diet for the optimal control of BP values.

## 9. Other Innovative Nutritional Approaches for Essential AH Treatment

Among the innovative nutritional approaches for AH, considerable interest was aroused by intermittent fasting (IF) [134], which is less restrictive when compared to CRD [135]. IF consists in the assumption of the normal daily caloric quote in a well-defined time gap, daily or weekly [136]. In the IF panorama, two basics scenarios are commonly used. The first one is time-restricted feeding (TRF), and it can be applied in three different alternatives: (i) 16/8, (ii) 18/6, or (iii) 20/4. For example, in the case of “16/8” the first value indicates 16 hours of fast, and 8 indicates the nutritional window. This scheme can be applied to the different variants [136]. An alternative to TRF is a 24-h fasting period (meaning caloric assumption of around 400-600 kcal/day) followed by a 24-h eating period twice or three times *per* week. In this scenario, there are two possible combinations: (i) 5/2 or (ii) 4/3. For instance, in the “5/2” plan caloric restriction is applied for two days, while normal diet is followed for five days [137].

The overview of the mechanisms that have been hypothesized to assess the correlation between IF and human health are the reduction of OS, improvement of cognition function, and anti-inflammatory effects. With regard to the cardioprotective effects, research has observed a reduction of adipose tissue (visceral); an increased concentration of adiponectin; a decrease of leptin and LDL-c; and prevention of CV diseases, particularly AH [138].

According to recent studies, it is assumed that there are mechanisms to assess the correlation between IF and human health [139], namely circadian rhythm (if disturbed, it can cause an energy imbalance and it means an enhanced risk of chronic non-communicable diseases onset [140]) and gut microbiota dysbiosis (if the microbiota is altered there is an impaired gut permeability, with the promotion of systemic inflammation [141,142,143]). Fasting regiments can also impact on modifiable CV risk factors such as energy intake [144], energy expenditure, [145] and sleep quality [146].

The IF diet has shown positive effects in lowering BP, with a recent study conducted by Erdem et al. [81] evaluating, in 60 subjects, the possible effects on BP values. The obtained results showed that IF significantly reduced 24-hour urinary sodium excretion, being associated with a decrease in SBP (*p* < 0.001) and DBP (*p* < 0.039) values. The authors concluded that this reduction is partly related to the decrease sodium consumption during the IF period.

Another interesting study was conducted by Wilhelmi de Toledo et al. [83] on 1422 subjects with one-year follow-up. IF period was comprised between 4 and 21 days, and the enrolled patients were instructed to perform a moderate-intensity physical exercise. Enrolled patients were grouped into four subgroups of 5, 10, 15, and 20 ± 2 days of IF. Every meal contained 200–250 kcal on average. The authors observed a decrease of SBP and DBP in patients who followed IF for a longer period of time; however, in this study, the authors did not specify the amount of salt introduced during the IF period and in particular whether there were any changes in its intake respect to the non-fasting period. They speculated that the drop of BP was due to the enhancement of parasympathetic nervous system activity, of norepinephrine excretion by the kidney and natriuretic peptides, and greater insulin sensitivity. Moreover, it has been observed that the positive effects induced by IF were limited to the nutritional intervention time; in fact, when the nutritional treatment was suspended, the BP values returned to their initial values [84]. To understand whether the reduction of the BP values during the IF are related to reduced caloric intake or reduced salt intake, further randomized clinical trials are needed to assess the impact of each variable on BP values.

During IF, the concentration of plasma glucose decreases. The glycogen reserves in the liver are consumed, and at the same time the gluconeogenesis process is activated. Insulin and IGF-1 levels are reduced in the bloodstream, while glucagon levels are increased. During the lipolysis process, fatty acids are released to be converted through beta oxidation, to be released in the blood and used as a source of energy [147]. An IF nutritional plan improves glucose metabolism even in patients with type 2 diabetes mellitus. As reported in a study conducted by Furmili et al. [82], after 12 weeks of almost 850 kcal daily meals, enrolled subjects showed a weight loss with a normalized fasting glycemia, reduced values of glycated hemoglobin (Hba1c) and increased insulin sensitivity [80]. 

In animal and in human studies, it has been showed that IF is able to improve physical function, moreover, in mice kept in an IF state, showed greater resistance to running. Other parameters such as balance and coordination were also positive [148].

## 10. Conclusions

In recent years, there has been a growing interest in identifying an alternative food strategy for achieving and maintaining weight loss in overweight people and to counteract the onset of CV disease. The high BP is one of the main risk factors of serious disabling diseases such as stroke, myocardial ischemia, and heart and kidney failure. In all AH forms, from the milder ones to those refractories to the pharmacotherapy, the correct nutritional approach turns out to be effective in order to reduce in a short period of time the BP values until the target range is obtained. The use of CRD, as an innovative nutritional-dietary approach, highlighted a significant reduction in BP values, an improvement in endothelial dysfunction, causing a decrease in metabolic and inflammatory parameters related to chronic non-communicable diseases. The results of the clinical intervention studies do not allow us to reach solid and definitive conclusions about the long-term efficacy and safety of this nutritional approach, especially in terms of the risk of developing side effects and nutritional inadequacy from lack of macro and micronutrients.

## Figures and Tables

**Table 1:** Dietary approaches in the arterial hypertension management.

Nutritional Treatment	Type of the Study	Authors	Year	Organ/System/Metabolic Target	Duration of Intervention	Impact of Intervention
CRD	Animal study	Zanetti et al. [64]	2010	Vascular endothelial Oxidative stress	3 weeks	↓ Oxidative stress ↓ iNOS ↓ Total nitrite ↓ Calcium-independent NOS activity ↑ SOD activity
CRD	Animal study	Rippe et al. [65]	2010	Vascular endothelial Oxidative stress	8 weeks	↓ Oxidative stress ↓ Blood glucose ↓ TG ↑ NO bioavailability ↑ Arterial expression of SIRT1
CRD	Animal study	Donato et al. [66]	2013	Oxidative stress Cardiovascular	30–31 months	↓ NADPH oxidase activity ↓ Oxidative stress ↓ TG ↓ BW ↑ SOD activity ↑ Catalase
CRD	Animal study	Kobara et al. [67]	2015	Cardiovascular Oxidative stress	4 weeks	↓ ROS ↓ Cardiac hypertrophy and fibrosis
CRD	Animal study	Waldman et al. [68]	2018	Cardiovascular Glucose profile	4 weeks	↓ Oxidative stress ↓ Inflammation ↑ SIRT1
CRD	Animal study	An et al. [69]	2020	Cardiovascular Iron metabolism	12 weeks	↓ Cardiac hypertrophy and fibrosis ↓ Cardiac inflammation ⇅ Cellular regulation of iron homeostasis
CRD	Human study	Wadden et al. [70]	1998	Leptin Body composition	40 weeks	↓ BW ↓ FM ↓ Serum leptin
CRD	Human study	Nakano et al. [71]	2001	Glucose profile ANS Body composition	2 weeks	↓ BMI ↓ BW ↓ TG ↓ HOMA-index ↓ SBP ↓ DBP
CRD	Human study	Facchini et al. [72]	2003	Cardiovascular Body composition	3 weeks	↓ BMI ↓ Heart rate ↑ Parasympathetic activity
CRD	Human study	Das et al. [73]	2007	Body composition Lipid profile	12 months	↓ FM ↓ BW ↓ TG ↓ insulin ↓ LDL-c ↓ TC ↑ HDL-c
CRD	Human study	Lecoultre et al. [74]	2011	Body composition Leptin	6 months	↓ BW ↓ Mean 24 h circulating leptin ↓ Urinary norepinephrine
CRD	Human study	Stewart et al. [75]	2013	Body composition Lipid profile	24 months	↓ LDL-c ↓ TC/HDL-c↓ TG ↓ DBP ↓ BW ↓ BMI ↓ FM ↓ MSS ↑ IS
CRD	Human study	Ruggenenti et al. [76]	2017	Renal function Glucose profile Body composition	6 months	↓ WC ↓ BW ↓ BMI ↓ BG ↓ HbA_1c_
CRD	Human study	Most et al. [77]	2018	Cardiovascular Glucose profile	24 months	↓ VAT ↓ SAAT ↓ SBP ↓ DBP ↓ TC ↓ LDL-c ↓ IR
CRD	Human study	Kraus et al. [78]	2019	Body composition Lipid profile Glucose profile Cardiovascular Lipidic profile	24 months	↓ LDL-c ↓ TC/HDL-c ↓ SBP ↓ DBP ↓ MSS ↑ IS
DASH DIET	Human study	Harsha et al. [79]	1999	Cardiovascular	8 weeks	↓ SBP ↓ DBP
IF	Human study	Arnason et al. [80]	2017	Glucose profile Body composition	6 weeks	↓ BW ↓ BMI
IF	Human study	Erdem et al. [81]	2018	Cardiovascular	-	↓ SBP ↓ Heart rate ↓ USE
IF	Human study	Furmli et al. [82]	2018	Glucose profile Body composition	12 weeks	↓ HbA_1c_ ↓ BW ↓ WC
IF	Human study	Wilhelmi de Toledo et al. [83]	2019	Cardiovascular Glucose profile Body composition Lipidic profile	24 months	↓ SBP ↓ DBP ↓ BW ↓ Abdominal circumference ↓ Blood glucose ↓ TG ↓ LDL-c ↓ HDL-c ↓ TC ↑ Physical and emotional well-being
CRD *vs* IF	Animal study	Magger et al. [84]	2006	Cardiovascular Body composition	16 weeks	↓ BW ↓ Heart rate ↓ SBP ↓ DBP ↓ Blood glucose

Abbreviations: ANS, autonomic nervous system; BG, blood glucose; BMI, body mass index; BW, body weight; CRD, caloric restriction diet; DBP, diastolic blood pressure; FM, fat mass; HbA_1c_, glycated hemoglobin; HDL-c, high-density lipoprotein cholesterol; IF, intermittent fasting; iNOS, inducible nitric oxide synthase; IR, insulin resistance; IS, insulin sensitivity; LDL-c, low density lipoprotein cholesterol; MSS, metabolic syndrome score; NADPH, nicotinamide adenine dinucleotide phosphate; NO, nitric oxide; ROS, reactive oxygen species; SAAT, subcutaneus abdominal adipose tissue; SBP, systolic blood pressure; SIRT1, sirtuin 1; SOD, superoxide dismutase; TC, total cholesterol; TG, triglycerides; USE, urinary sodium excretion; VAT, visceral adipose tissue; WC, waist circumference, ↑ increase, ↓ decrease.

## Data Availability

Not applicable.

## List of Acronyms

ACE	angiotensin conversion enzyme
AH	arterial hypertension
AMPK	AMP-activated protein kinase
ARB	angiotensin receptor blockers
AS	arterial stiffness
BMI	body mass index
BP	blood pressure
BW	body weight
CALERIE	Comprehensive Assessment of Long-term Effects of Reducing Intake of Energy
cBP	central blood pressure
cGMP	cyclic guanosine monophosphate
CKD	chronic kidney disease
CRD	caloric restriction diet
CRP	c-reactive protein
CV	cardiovascular diseases
DBP	diastolic blood pressure
eNOS	endothelial nitric oxide synthase
ESC	European Society of Cardiology
ESH	European Society of Hypertension
HDL-c	high-density lipoprotein cholesterol
HRV	heart rate variability
IF	intermittent fasting
IGF-1	insulin-like growth factor-1
IL	interleukin
LDL-c	low-density lipoprotein cholesterol
LVH	left ventricular hypertrophy
m-TOR	mammalian target of rapamycin
MBIP	model-based image processing
MDRD	modification of diet in real disease study
NAD	nicotinamide dinucleotide
NAS	nephroangiosclerosis
NIH	National Institute of Health
NO	nitric oxide
NOS	nitric oxide-synthase
OS	oxidative stress
PGC1-α	peroxisome proliferator-activated receptor gamma coactivator 1-α
PWV	pulsating wave velocity
RAAS	renin–angiotensin–aldosterone system
ROS	reactive oxygen species
SBP	systolic bloody pressure
SIRT1	sirtuin1
SOD	superoxide dismutase
TGF- β1	transforming growth factor-β1
TNF	tumor necrosis factor
TRF	time-restricted feeding
UAE	urinary albumin excretion
WHO	World Health Organization
